# Computing Viscosities of Mixtures of Ester-Based Lubricants at Different Temperatures

**DOI:** 10.1021/acs.jpcb.2c08553

**Published:** 2023-03-08

**Authors:** Davide Sarpa, Dimitrios Mathas, Vasilios Bakolas, Joanna Procelewska, Joerg Franke, Martin Busch, Philipp Roedel, Christof Bohnert, Marcus Wolf, Chris-Kriton Skylaris

**Affiliations:** † Department of Chemistry, University of Southampton, Highfield, University Road, Southampton SO17 1BJ, U.K.; ‡ Schaeffler Technologies AG & Co. KG, Industriestraße 1-3 91074, Herzogenaurach, Germany

## Abstract

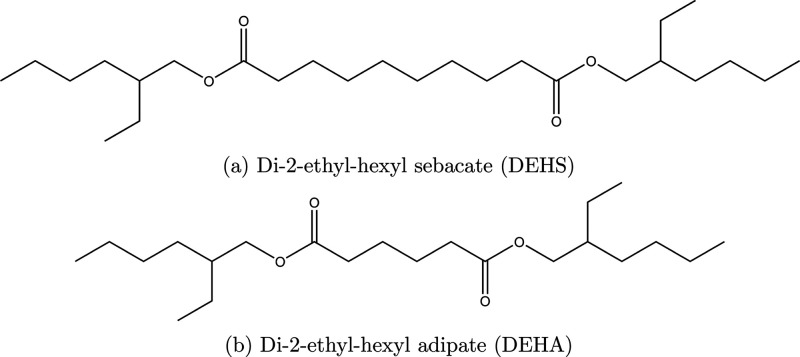

Synthetic esters are used as lubricants for applications at high temperatures, but their development can be a trial and error process. In this context, molecular dynamics simulations could be used as a tool to investigate the properties of new lubricants, in particular viscosity. We employ nonequilibrium molecular dynamics (NEMD) simulations to predict bulk Newtonian viscosities of a set of mixtures of two esters, di(2-ethylhexyl) sebacate (DEHS) and di(2-ethylhexyl) adipate (DEHA) at 293 and 343 K as well as equilibrium molecular dynamics (EMD) and NEMD at 393 K and compare these to experimental measurements. The simulations predict mixture densities within 5% of the experimental values, and we are able to retrieve between 99% and 75% of the experimental viscosities for all ranges of temperature. Experimental viscosities show a linear trend which we are able to capture using NEMD at low temperature and EMD at high temperature. Our work shows that, using EMD and NEMD simulations, and the workflows we developed, we can obtain reliable estimates of the viscosities of mixtures of industrially relevant ester-based lubricants at different temperatures.

## 1 Introduction

Lubricants are an essential tool to reduce machine degradation and improve efficiency by reducing the friction between surfaces.^1^ Lubricants can be divided into biological and nonbiological, the first type is required where contamination must be kept as low as possible as in the food or drug industry. Nonbiological lubricants are divided into two main categories: mineral oils and synthetic oils and are the most widespread types of lubricants.^2^ Mineral oils are cheap and the most common in many applications, but they are made from petroleum which leads to very complex formulations made by more than 30 different molecules. This makes it hard to develop new lubricants or to improve their performances.^1^ Synthetic oils, on the other hand, are more expensive, and they were developed mainly to work in extreme conditions such as high temperatures or high pressures.^3^

Lubricant development is an important field of research in tribology, but due to lubricant complexity, the process is mostly trial and error based and not much is known about the atomistic behavior.^1^ We need a better approach to predict viscosity and understand the behavior at a microscopic scale. In this setting, molecular dynamics (MD) simulations have come forward as a tool to predict static and dynamic properties such as density or viscosity for a range of lubricants providing useful insights into the chemical and physical behavior of such complex systems.

Viscosity is one of the most important properties to study because it is directly connected to the friction reduction behavior and lubrication regime. It is mainly affected by lubricant composition, temperature, pressure, and shearing. In the last 40 years, equilibrium (EMD) and nonequilibrium (NEMD) molecular dynamics simulations have been carried out by different researchers. Allen et al.^4^ showed how the density is affected mainly by intermolecular interactions while the torsional part of the force field and its coupling to the translational degrees of freedom affect the viscosity. They show that this coupling increases with the chain length and that the time needed for the Green–Kubo viscosity integral to convergence can be roughly estimated by the rotational relaxation time, which increases with chain length. This finding was also confirmed by other authors.^5,6^ The effects of temperature, pressure, and other thermodynamic properties on viscosity have been studied by various researchers.^7−13^ Molecular dynamics was employed on different systems including glass, polyethers, ionic liquids, and biolubricants;^14−18^ force field impact was also studied.^19,20^ Lacks et al. showed that, if the simulations sample a phase space local minima, it may lead to having a lower or higher shear rate than expected, and that would influence the value of the viscosity and suggest the importance of averaging over multiple trajectories. This provides a simple yet powerful methodology to solve this problem.^21^ Lin and co-workers showed the predictive power of nonequilibrium molecular dynamics for a polyol system at different temperatures and pressures.^22^ Equilibrium and nonequilibrium should provide the same results in the limit of zero shear, but the latter is the most appropriate for longer, flexible molecules^5,6^ while the former works best for low viscosity fluids, lower than 20 mPa s.^23^

Numerous detailed reviews are available on the use of molecular dynamics in tribology simulations, and the reader should refer to these for more information on the field.^24−26^

**Figure 1:**
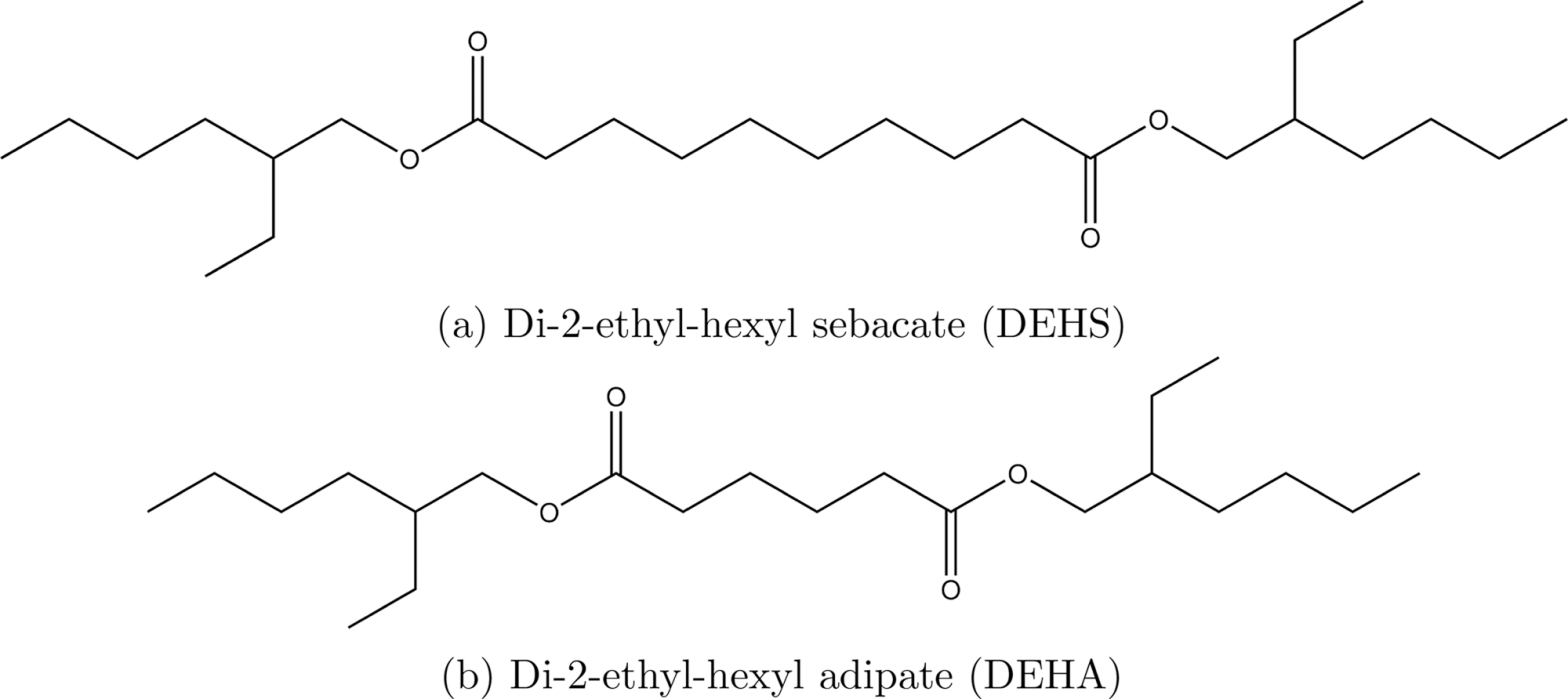
Structures of two esters, sebacate (top) and adipate (bottom), studied in this work.

In our group, we have previously studied the effect of pressure, temperature and force field on an 9,10-dimethyloctadecane system which was studied as a model of PAO-2 lubricant as it is one of the main components.^13^ We then decided to focus on more realistic yet simple systems as the mixture of two synthetic esters, di-2-ethylhexyl sebacate (DEHS) (Figure 1a) and di-2-ethylhexyl adipate (DEHA) (Figure 1b). They are industrially relevant lubricants, and their applications involve high temperatures and/or pressures.^27−34^ In addition, most molecular dynamics simulations of mixtures consist of linear, branched alkanes or fatty acids.^18,35,36^ To the best of our knowledge there are no molecular dynamics simulations for ester mixtures in the literature. Our goal is to provide a workflow for ester mixtures that allow a reliable and accurate viscosity prediction that could lead to a better understanding and design of future ester-based lubricants. We evaluated densities and viscosities via experiments and NEMD simulations at two different temperatures, 293 and 343 K, and via experiments, NEMD, and equilibrium molecular dynamics (EMD) at 393 K, on a set of mixtures: DEHS and DEHA samples were blended in 10% steps from 100% DEHS up to 100% DEHA for a total of 11 different mixtures.

In section 2, methodology and computational and experimental procedures are explained, in section 3, main results and their discussion are presented, and in section 4, conclusions are presented.

## 2 Methodology

### 2.1 Equilibrium Molecular Dynamics

Molecular dynamics is a simulation technique used to solve Newton’s equations of motion for a collection of interacting particles. The interactions are modeled via an empirical force field for which the general form is
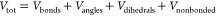
1Many algorithms are available to solve the equations of motion; in this work the velocity Verlet algorithm was used.^37^ In equilibrium molecular dynamics, it is possible to compute transport properties by making use of the Green–Kubo formulas that relate transport properties with correlation functions of the system;^38^ for viscosity it is equal to
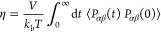
2where *k*_*b*_*T* is the Boltzmann constant, *T* is the temperature, and the integrand is the autocorrelation function of the off-diagonal components of the pressure tensor.

The convergence of the integral depends on the simulation time as well as the decay of the autocorrelation function.

### 2.2 Nonequilibrium Molecular Dynamics

Nonequilibrium molecular dynamics is a simulation technique in which a perturbation is applied to the system to closely represent real-world applications. Nonequilibrium MD implements a new algorithm called SLLOD^39,40^ which applies a streaming velocity by introducing a fictitious external field in the equations of motion:
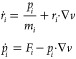
3where ∇*v* is the streaming velocity. In lubricant simulations, we are mainly interested in planar shear flow for which the streaming velocity is equal to
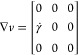
4where  is the shear rate or the magnitude of the velocity gradient.

These new equations have to be used with a set of proper periodic boundary conditions (pbc) for a planar shear rate. The appropriate pbc are the Lee–Edwards periodic boundary condition or the Lagrangian–Rhomboid.^40^ The equations of motion are further modified by introducing thermostats and/or barostats. In particular, the Nosé–Hoover thermostat and barostat were used in this study to maintain constant pressure and temperature.^41,42^

In NEMD simulations, it is possible to directly calculate the viscosity by
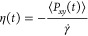
5where ⟨*P*_*xy*_(*t*)⟩ is the average of the off-diagonal term of the pressure tensor and  is the shear rate.

System size, long-range cutoff, time step, shear rate, and simulation length were investigated via trial and error process.

### 2.3 Computational Details

#### 2.3.1 System Setting

A set of 11 mixtures with different concentrations of DEHS and DEHA were prepared using Packmol^43^ to generate starting configurations which were then used by Moltemplate^44^ to generate the files necessary to run the simulations using the molecular dynamics software LAMMPS.^45^

Each system consists of ≈12000 atoms (the numbers of DEHS and DEHA molecules per mixture are available in Table S1), and the force field employed throughout this work is the L-OPLS-AA^46^ with a long-range cutoff of 12 Å. The reciprocal space part is solved using the PPPM algorithm^47^ with a relative error in forces of 1 × 10^–4^. All systems were run using 1 fs as the time step.

#### 2.3.2 Equilibration

Each system first underwent a minimization step with energy and force thresholds of 1 × 10^–5^ kcal/mol and 1 × 10^–7^ kcal/mol/Å, respectively, and then went through a 5 ns *NVT* run with a temperature damping parameter of 100 fs to reach the target temperature of 293, 343, or 393 K. A 20 ns *NPT* run was performed to allow the system to relax its volume and reach the target pressure of 1 atm. Another 5 ns *NPT* simulation was also run, and the average density was calculated. The system was then scaled to match the average density as in ref (22); then the systems were allowed to relax with another 5 ns *NVT* simulation. This was the starting point for both EMD and NEMD following different workflows.

#### 2.3.3 EMD Simulations

For the EMD only the system at 393 K was studied and the system underwent a 5 ns *NVT* simulation, at the end of which five configurations of positions and velocities were saved 50 fs apart from each other. These 5 different data files were used to run the EMD production runs which consisted of an 80 ns *NVT* run; the autocorrelation and viscosity were calculated on the fly via fixes available in LAMMPS as an average of 3 off-diagonal pressure tensor values, *P*_*xy*_, *P*_*yz*_, *P*_*xz*_. The viscosity reported in this study is the 5 trajectory average.

#### 2.3.4 NEMD Simulations

**Figure 2:**
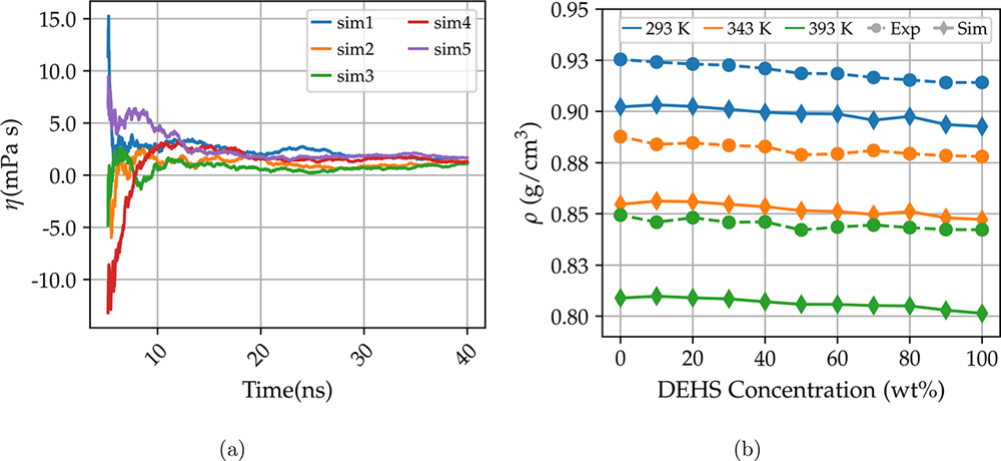
Viscosity values from 5 different trajectories for the 10% DEHS 90% DEHA mixture at 343 K (left), Experimental and simulated densities (right) in g cm^–3^ as a function of DEHS concentration (wt %) at 293, 343, and 393 K. Circles are experimental data; diamonds are simulated data.

The system was sheared for 10 ns using a *NVT*/sllod run with a shear rate of 1 × 10^8^ s^–1^, at the end of which five configurations of positions and velocities were saved 50 fs apart from each other. These data files will be used as a starting point for the production run, generating in total 5 different trajectories per mixture where the final viscosity will be the average of trajectory viscosities (Figure 2a). The production run consists of 40 ns using a *NVT*/sllod run with a shear rate of 1 × 10^8^ s^–1^. The viscosity for each trajectory was calculated using the last 35 ns. The velocity profile was checked to be linear for all trajectories as expected from bulk shear rate lubricant simulations.

### 2.4 Experimental Procedure

The dynamic viscosities and the densities of DEHS and DEHA mixtures were measured by using an Anton Paar Stabinger viscometer, Stabinger SVM 3001, with autosampler XSample 530.

The Stabinger viscosity measurement principle is based on a floating cylinder in test fluid which is centered by a rotating force. This is tracked in terms of speed and torque as viscosity indicators by a magnetic field and retarder, avoiding any bearing friction. Density is captured by the principle of a bending oscillator. The measurements could be done in an effective range from 0.2 up to 30000 mm^2^/s. Based on dynamic viscosity and density the kinematic viscosity, ν, could be calculated as
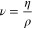
6where ν is the dynamic viscosity and ρ is the density.

The shear rate is part of the measurement principle and depends on the measured viscosity; hence, it could not be preselected. Shear rate value in experimental measurements is at most in the range of 1000 s^–1^. This could be considered as low shear compared to bearing applications, but the value is comparable to measuring methods such as Ubbelohde. DEHS and DEHA samples were blended in 10% steps from 100% DEHS up to 100% DEHA. The measurements were done under normal pressures and covered three temperatures (293, 343, and 393 K) for every mixture. The densities and viscosities reported are an average of 2 and 3 different measurements, respectively.

## 3 Results and Discussion

### 3.1 Experimental Results

Table 1 reports the measured densities in g cm^–3^ and measured viscosities in mPa s at 293, 343, and 393 K. All measurements were obtained at atmospheric pressure.

**Table 1:** Experimental Densities, ρ, in g cm^–3^, and Dynamic Viscosities, η, in mPa s, at 293, 343, and 393 K for the 11 Mixtures Studied

Mixture (wt %)	Density (293 K)	Density (343 K)	Density (393 K)	Viscosity (293 K)	Viscosity (343 K)	Viscosity (393 K)
100% DEHS 0% DEHA	0.9141	0.8779	0.8421	21.53	4.86	2.08
90% DEHS 10% DEHA	0.9141	0.8781	0.8418	21.14	4.82	2.07
80% DEHS 20% DEHA	0.9153	0.8791	0.8429	20.22	4.66	2.01
70% DEHS 30% DEHA	0.9166	0.8813	0.8446	19.35	4.33	1.91
60% DEHS 40% DEHA	0.9185	0.8813	0.8461	18.10	4.31	1.83
50% DEHS 50% DEHA	0.9186	0.8825	0.8471	17.70	4.12	1.76
40% DEHS 60% DEHA	0.92105	0.8838	0.8468	16.36	3.96	1.75
30% DEHS 70% DEHA	0.9226	0.8847	0.8474	15.46	3.82	1.70
20% DEHS 80% DEHA	0.9232	0.8857	0.8484	14.97	3.68	1.64
10% DEHS 90% DEHA	0.9241	0.8868	0.8492	14.41	3.54	1.59
0% DEHA 100% DEHA	0.9254	0.8877	0.8488	13.72	3.43	1.54

The simulated and experimental densities for the mixtures were plotted in Figure 2b, as a function of DEHS concentration. The densities decrease as the temperature increases, as expected.

**Figure 3:**
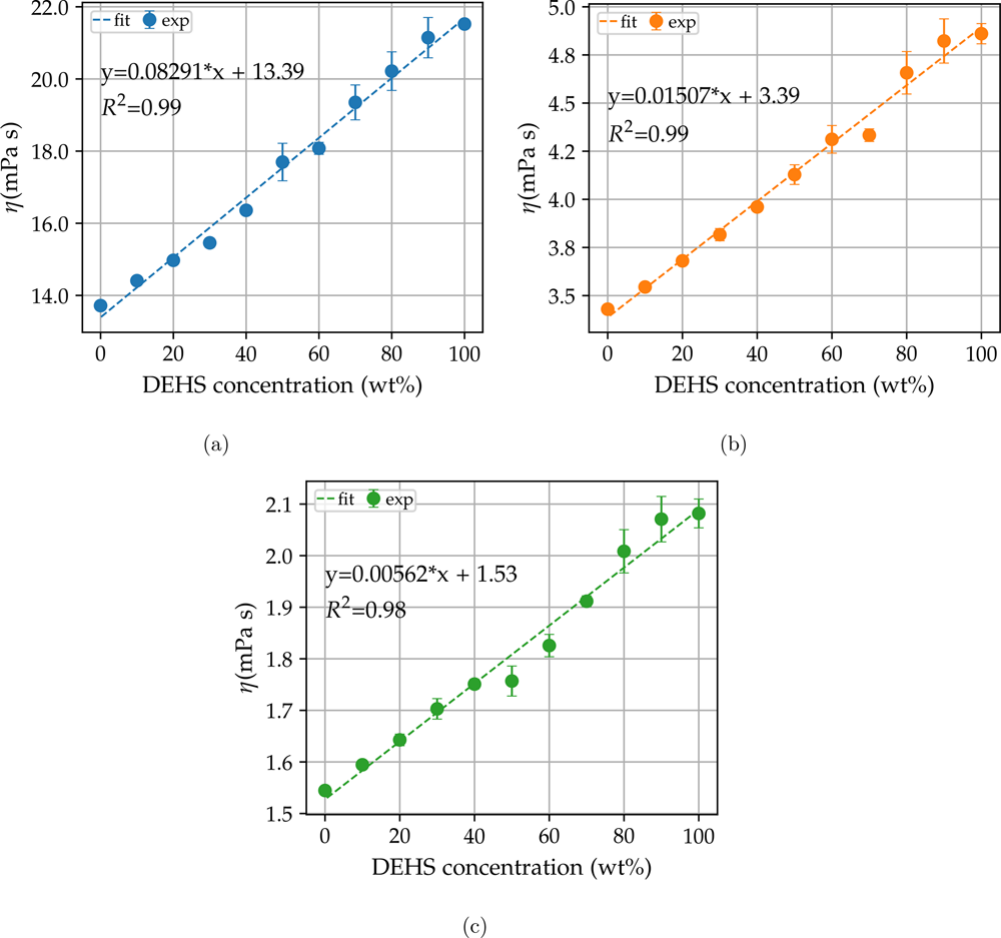
Experimental data and linear fit as a function of DEHS concentration at 293 K (top left, blue), 343 K (top right, orange), and 393 K (bottom, green). The slope intercepts equation and *R*^2^ are reported in the plot.

Figure 3 plots the measured viscosities as a function of DEHS concentration at 293 K (Figure 3a), 343 K (Figure 3b), and 393 K (Figure 3c). We decided to fit the viscosity data to a linear function:
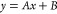
7where *x* is the concentration by weight of DEHS in the mixtures. The viscosities change linearly with mixture composition at all temperatures. The *B* values correspond to the viscosities of pure DEHA, and the *A* corresponds to the slopes of the fit.

The standard deviations for the viscosity measurements are reported as percentages of the mean and are on average 1.25% for the systems at 293 K, 1.04% at 343 K, and 1.08% at 393 K.

### 3.2 Simulation Results

#### 3.2.1 NEMD

We performed NEMD simulations on three different temperatures to be compared to experimental results. Density values are reported in Table 2 while viscosities are shown in Table 3.

**Table 2:** Densities ρ, in g cm^–3^, Obtained from NEMD 40 ns Simulations with a Shear Rate of 1 × 10^8^ s^–1^ at 293, 343, and 393 K for the 11 Mixtures Studied

Mixture	293 K	343 K	393 K
100% DEHS	0.8926	0.8472	0.8014
90% DEHS 10% DEHA	0.8936	0.8480	0.8029
80% DEHS 20% DEHA	0.8975	0.8511	0.8050
70% DEHS 30% DEHA	0.8957	0.8497	0.8052
60% DEHS 40% DEHA	0.8987	0.8511	0.8058
50% DEHS 50% DEHA	0.8989	0.8515	0.8058
40% DEHS 60% DEHA	0.8995	0.8535	0.8071
30% DEHS 70% DEHA	0.9011	0.8547	0.8085
20% DEHS 80% DEHA	0.9025	0.8510	0.8090
10% DEHS 90% DEHA	0.9032	0.8563	0.8098
100% DEHA	0.9023	0.8547	0.8089

**Table 3:** Viscosities η, in mPa s, Obtained from NEMD 40 ns Simulations with a Shear Rate of 1 × 10^8^ s^–1^ at 293, 343, and 393 K for the 11 Mixtures Studied^a^

Mixture	293 K	343 K	393 K
100% DEHS 0% DEHA	19.35 ± 1.00 (5.2%)	3.78 ± 0.89 (23.5%)	2.07 ± 0.38 (18.4%)
90% DEHS 10% DEHA	17.30 ± 0.91 (5.3%)	3.94 ± 0.24 (6.0%)	1.40 ± 0.36 (26.7%)
80% DEHS 20% DEHA	16.86 ± 2.43 (14.4%)	3.57 ± 0.61 (17.1%)	1.48 ± 0.36 (24.3%)
70% DEHS 30% DEHA	15.38 ± 2.0 (13.0%)	2.74 ± 0.85 (31.0%)	1.51 ± 0.62 (41.0%)
60% DEHS 40% DEHA	15.18 ± 1.08 (7.1%)	2.71 ± 0.53 (19.6%)	1.55 ± 0.30 (19.3%)
50% DEHS 50% DEHA	14.53 ± 1.29 (8.87%)	2.80 ± 0.84 (30.0%)	1.44 ± 0.35 (24.3%)
40% DEHS 60% DEHA	13.71 ± 1.47 (10.7%)	3.72 ± 0.74 (19.9%)	1.67 ± 0.38 (22.7%)
30% DEHS 70% DEHA	13.33 ± 1.13 (8.48%)	3.68 ± 0.22 (6.0%)	1.14 ± 0.36 (31.6%)
20% DEHS 80% DEHA	12.89 ± 0.80 (6.2%)	2.79 ± 0.53 (18.9%)	1.37 ± 0.34 (24.8%)
10% DEHS 90% DEHA	12.11 ± 1.59 (13.1%)	3.11 ± 0.42 (13.5%)	1.25 ± 0.44 (35.2%)
0% DEHA 100% DEHA	11.90 ± 0.5 (4.2%)	3.24 ± 0.67 (20.8%)	1.26 ± 0.29 (23.0%)

a Standard deviations reflect the spread of the trajectories.

The viscosities reported are trajectory averages (Figure 2a), and the standard deviation reflects the spread of those trajectories. The number of trajectories used to calculate the average was selected by looking at the individual trajectories to check if any of them was trapped in local minima, a region of lower or higher viscosity than expected.^21^

In Figure 2, we plot (right) both the simulated and experimental densities as a function of DEHS concentration. The simulated densities show, for all temperatures, a decreasing trend in values. At 293 K simulated densities are on average lower than experimental ones by 2.13%, at 343 K by 3.11%, and at 393 K by 4.3%. L-OPLS-AA underestimates densities for all temperatures. The simulations get less accurate as the temperature increases, which is in agreement with another similar study.^22^

**Figure 4:**
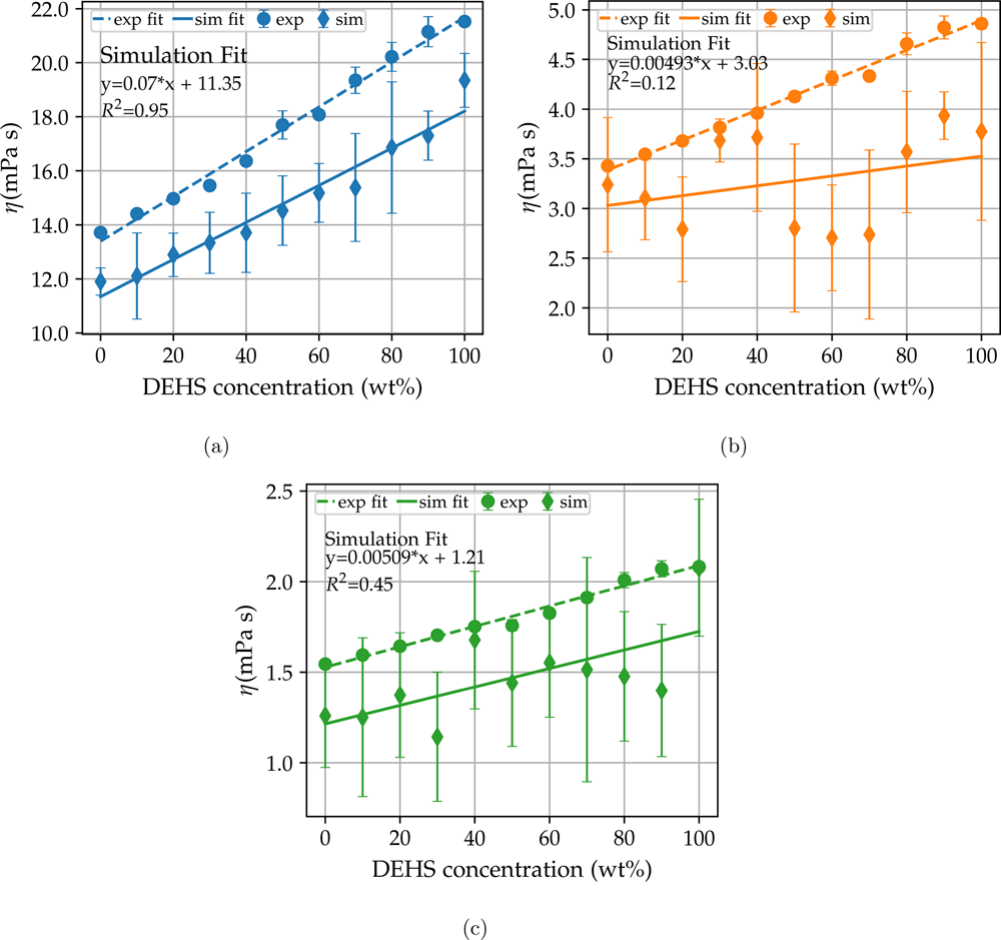
Simulation and experimental fit for three temperatures 293, 343, and 393 K. Dashed lines and circles refer to experimental fit and values, and full lines and diamonds refer to simulated values. Error bars reflect the spread of trajectories for simulated values.

Simulation viscosities were compared to the experimental measurements, and all values are, on average, lower than their respective experimental viscosities by 17.4% and no higher than 25%, which is in agreement with values from a study on a closely related polyol system at different pressures and temperatures.^22^ We applied the same fitting function to the simulated values, and the linear behavior is obtained only in the simulation at 293 K with *R*^2^ = 0.95; the slope is comparable to the experimental value, but the viscosities are on average underpredicted by 2 mPa s. The situation gets worse for the simulations at 343 K with *R*^2^ = 0.12 and at 393 K with *R*^2^ = 0.45. Increasing the temperature from 293 K (Figure 4a) to 343 K (Figure 4b) and 393 K (Figure 4b) decreases the viscosity exponentially, but the spread between trajectories does not decrease. This could lead to problems with the detection of possible linear or nonlinear relations in viscosity simulations at high temperatures, or more generally, this could be the case for low viscosity lubricants.

The underprediction of viscosities could be linked to the force field limitations or the use of a profile-biased thermostat which assumes a velocity linear profile.^40^

To decrease the spread of the trajectories at 393 K, we decided to run new EMD and NEMD simulations for up to 120 ns starting with three mixtures (100% DEHA, 100% DEHS, and 50% DEHS 50% DEHA) due to being the end and the middle point of the line plot and providing guidance whether running for a longer time would decrease the spread.

Table 4 shows that increasing the simulation length decreases the spread between trajectories for both NEMD and EMD, and it shows that NEMD seems to be less precise than EMD.

**Table 4:** Experimental and EMD and NEMD Simulations for 3 Mixtures at 393 K^a^

Mixture	Exp	EMD (80 ns)	NEMD (80 ns)	EMD (120 ns)	NEMD (120 ns)
100% DEHS	2.05	1.41 ± 0.1	1.24 ± 0.34	1.46 ± 0.08	1.33 ± 0.13
50% DEHA 50% DEHS	1.79	1.23 ± 0.15	1.62 ± 0.45	1.24 ± 0.09	1.54 ± 0.24
100% DEHA	1.54	1.19 ± 0.08	1.11 ± 0.25	1.16 ± 0.11	1.14 ± 0.22

a Simulations are reported for two different simulation lengths: 80 and 120 ns. Viscosities reported are trajectory averages.

The error or spread for NEMD at 120 ns is higher than that for EMD, and it is still higher or at least comparable to that for EMD at 80 ns. We decided to run EMD on the rest of the mixtures at 393 K for 80 ns as it appears to be a good trade-off between computational cost and precision.

**Figure 5:**
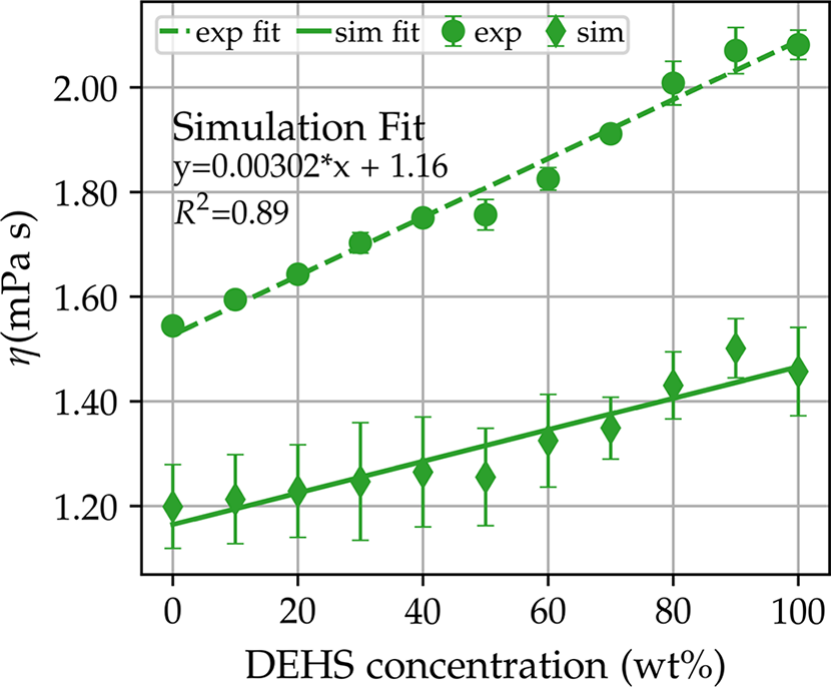
Equilibrium simulation and experimental fit for mixtures at 393 K. Dashed lines and circles refer to experimental fit and values; full lines and diamonds refer to simulated values. Error bars are given for simulated values.

Figure 5 shows the line fit for the EMD simulations; the linearity is more robust compared to the NEMD, with *R*^2^ = 0.88 for EMD against *R*^2^ = 0.45 for NEMD, which shows that the spread of trajectories played a big role in the noisier NEMD simulations and consequently in the linear fit. EMD can predict the linear trend qualitatively, but the slope and intercept are still off from the experiments.

We have run NEMD and EMD simulations for 11 different mixtures of esters at three different temperatures and compared them against experiments. We were able to retrieve between 99% and 75% of the experimental values, but the general viscosity trend was retrieved only for the lowest temperature due to the high spread between trajectories. We then compared EMD and NEMD for three mixtures to reduce the spread and EMD proved to be more precise, as it is able to reproduce, at least qualitatively, the linear behavior at high temperature. This could be related to the low viscosity values. In our study, the difference in viscosity between mixtures with a 10% increase of DEHS is, on average, 0.05 mPa s. This difference would require the methods and force field to be able to predict viscosities with a resolution of <0.05 mPa s to provide a quantitative description via a linear fit. All the raw viscosity and density data are provided in the Supporting Information.

### 3.3 Radial Distribution Function

**Figure 6:**
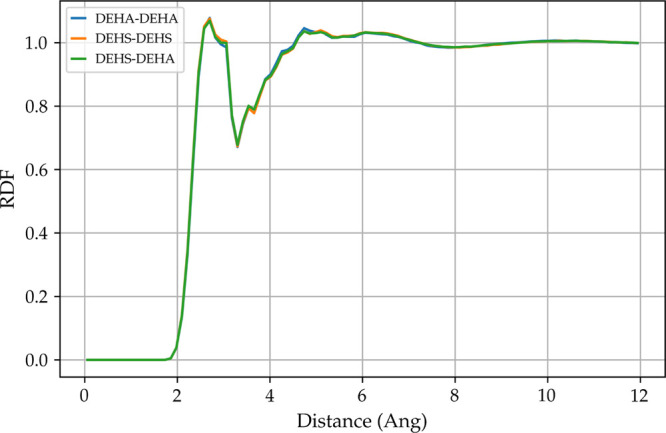
All-atom to all-atom radial distribution function for DEHS–DEHS, DEHA–DEHA, and DEHS–DEHA at 393 K.

The observed linearity in the viscosity plot (Figure 4) could be caused by the molecules having a similar structure, giving the liquid the properties of an ideal mixture. We can check this hypothesis by plotting a radial distribution function (rdf) among DEHS–DEHS, DEHA–DEHA, and DEHS–DEHA. If the mixture is ideal, then we would expect the rdf plots to be similar. Figure 6 shows the nonequilibrium all-atom rdfs for the 50% DEHS 50% DEHA mixture at 393 K. As expected, we observe that the DEHS–DEHS, DEHA–DEHA, and DEHS–DEHA rdfs are very similar. We can therefore conclude that linear viscosity behavior is caused by the formation of an ideal mixture.

**Figure 7:**
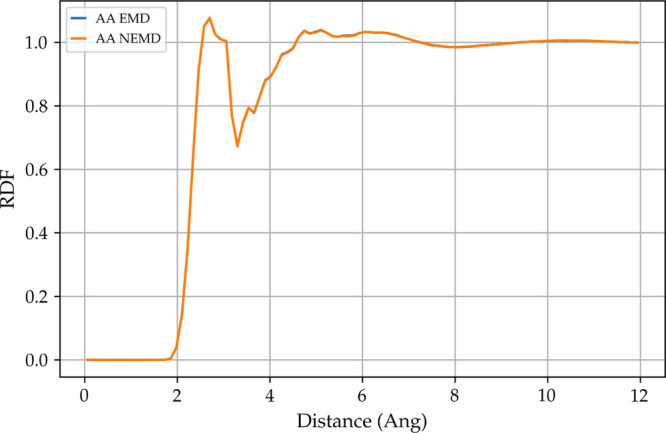
All-atom to all-atom radial distribution functions for 50% DEHS 50% DEHA simulation for both equilibrium and nonequilibrium molecular dynamics.

We have been underestimating the viscosity of this mixture. A possible cause of this underprediction would be a high shear rate that causes the fluid to transition to non-Newtonian behavior, specifically the so-called shear-thinning behavior where the viscosity decreases as the shear rate increases. Bernardino and Ribeiro^48^ showed that, for 1-ethyl-3-methylimidazolium based ionic liquid, a small change between the rdf of the NEMD simulation compared to the EMD is shear influenced and their fluid is in a shear-thinning region. Figure 7 shows the all-atom rdf for 50% DEHS 50% DEHA at 393 K for both NEMD and EMD where we observe perfect agreement. This allows us to assess the Newtonian regime of the NEMD simulation.

## 4 Conclusions

We performed experiments and nonequilibrium molecular dynamics (MD) 40 ns simulations on 11 different mixtures of two synthetic diesters at 293 and 343 K with a shear rate of 1 × 10^8^ s^–1^ and equilibrium and nonequilibrium MD simulations at 393 K for up to 120 ns. We employed a 5 trajectory average to avoid a skewed viscosity in case the system is stuck in local minima. We showed that the experimental viscosities of mixtures follow a linear trend for all temperatures, but only the simulations at 293 K were able to show the same linear trend via NEMD while simulations at 343 and 393 K did not show a trend. To solve this issue, we ran NEMD and EMD simulations at 393 K and the latter were able to achieve a low enough spread which led to the prediction of the expected linear trend, at a qualitative level. This is probably due to the small differences in viscosity between the mixtures, as such accuracy is beyond what can be obtained via MD and force fields. Viscosity values were overall underpredicted but were able to retrieve between 99% and 75% of the experimental value, the underestimation of viscosity could be caused by limitations of the force field or in the use of profile-biased thermostats which assume a linear velocity profile. Possible solutions both to underestimation and to the possibility of reproducing experimental trends would be to include viscosity during the force field parametrization and to employ configurational thermostats, but these developments are not available in commonly used simulation packages. In conclusion, NEMD simulations and EMD simulations using L-OPLS-AA and employing a 5 trajectory average predicted viscosity between 99% and 75% of experimental values. We expect that this study will serve as a basis for future simulations of mixtures of industrially relevant ester-based lubricants at different temperatures.

## Supporting Information Available

Number of DEHS and DEHA molecules (Table S1), experimental densities (Tables S2–S4), experimental viscosities (Tables S5–S7), NEMD viscosities (Tables S8, S10, S11), and EMD viscosities (Table S9) (PDF)

## References

[ref1] Mang T.; Dresel W. Lubricants and Lubrications, 2nd completely revised and extended; Wiley-VCH: Weinheim, 2007.

[ref2] Stachowiak G.; Batchelor A. Engineering Tribology; Elsevier: 2014; pp 51–104.

[ref3] Rudnick L. R. Synthetics, Mineral Oils, and Bio-Based Lubricants: Chemistry and Technology, 3rd ed.; CRC Press: Boca Raton, 2020; 1194 pp.

[ref4] Allen W.; Rowley R. L. Predicting the Viscosity of Alkanes Using Nonequilibrium Molecular Dynamics: Evaluation of Intermolecular Potential Models. J. Chem. Phys. 1997, 106, 10273–10281. 10.1063/1.474052.

[ref5] Cui S. T.; Cummings P. T.; Cochran H. D. The Calculation of Viscosity of Liquid N-Decane and n-Hexadecane by the Green–Kubo Method. Mol. Phys. 1998, 93 (1), 117–122. 10.1080/002689798169500.

[ref6] Mondello M.; Grest G. S. Viscosity Calculations of N-Alkanes by Equilibrium Molecular Dynamics. J. Chem. Phys. 1997, 106, 9327–9336. 10.1063/1.474002.

[ref7] McCabe C.; Cui S.; Cummings P. T. Characterizing the Viscosity–Temperature Dependence of Lubricants by Molecular Simulation. Fluid Phase Equilib. 2001, 183–184, 363–370. 10.1016/S0378-3812(01)00448-4.

[ref8] McCabe C.; Cui S.; Cummings P. T.; Gordon P. A.; Saeger R. B. Examining the Rheology of 9-Octylheptadecane to Giga-Pascal Pressures. J. Chem. Phys. 2001, 114, 1887–1891. 10.1063/1.1334676.

[ref9] Kondratyuk N.; Lenev D.; Pisarev V. Transport Coefficients of Model Lubricants up to 400 MPa from Molecular Dynamics. J. Chem. Phys. 2020, 152, 191104 10.1063/5.0008907. 33687262

[ref10] Kondratyuk N. D.; Pisarev V. V. Calculation of Viscosities of Branched Alkanes from 0.1 to 1000 MPa by Molecular Dynamics Methods Using COMPASS Force Field. Fluid Phase Equilib. 2019, 498, 151–159. 10.1016/j.fluid.2019.06.023.

[ref11] Ramasamy U. S.; Bair S.; Martini A. Predicting Pressure–Viscosity Behavior from Ambient Viscosity and Compressibility: Challenges and Opportunities. Tribol Lett. 2015, 57, 11 10.1007/s11249-014-0454-5.

[ref12] Tseng H.-C.; Wu J.-S.; Chang R.-Y. Shear Thinning and Shear Dilatancy of Liquid NHexadecane via Equilibrium and Nonequilibrium Molecular Dynamics Simulations: Temperature, Pressure, and Density Effects. J. Chem. Phys. 2008, 129, 014502 10.1063/1.2943314. 18624478

[ref13] Mathas D.; Holweger W.; Wolf M.; Bohnert C.; Bakolas V.; Procelewska J.; Wang L.; Bair S.; Skylaris C.-K. Evaluation of Methods for Viscosity Simulations of Lubricants at Different Temperatures and Pressures: A Case Study on PAO-2. Tribol. Trans. 2021, 64, 1138–1148. 10.1080/10402004.2021.1922790.

[ref14] Galamba N.; Nieto de Castro C. A.; Ely J. F. Shear Viscosity of Molten Alkali Halides from Equilibrium and Nonequilibrium Molecular-Dynamics Simulations. J. Chem. Phys. 2005, 122, 224501 10.1063/1.1924706. 15974685

[ref15] Borodin O.; Smith G. D.; Kim H. Viscosity of a Room Temperature Ionic Liquid: Predictions from Nonequilibrium and Equilibrium Molecular Dynamics Simulations. J. Phys. Chem. B 2009, 113, 4771–4774. 10.1021/jp810016e. 19275203

[ref16] Guo Q.; Chung P. S.; Chen H.; Jhon M. S. Molecular Rheology of Perfluoropolyether Lubricant via Nonequilibrium Molecular Dynamics Simulation. J. Appl. Phys. 2006, 99, 08N105 10.1063/1.2171937.

[ref17] Jadhao V.; Robbins M. O. Probing Large Viscosities in Glass-Formers with Nonequilibrium Simulations. Proc. Natl. Acad. Sci. U. S. A. 2017, 114, 7952–7957. 10.1073/pnas.1705978114. 28696320 PMC5544323

[ref18] Sneha E.; Revikumar A.; singh J. Y.; Thampi A. D.; Rani S. Viscosity Prediction of Pongamia Pinnata (Karanja) Oil by Molecular Dynamics Simulation Using GAFF and OPLS Force Field. Journal of Molecular Graphics and Modelling 2020, 101, 107764 10.1016/j.jmgm.2020.107764. 33032203

[ref19] Ewen J. P.; Gattinoni C.; Thakkar F. M.; Morgan N.; Spikes H. A.; Dini D. A Comparison of Classical Force-Fields for Molecular Dynamics Simulations of Lubricants. Materials 2016, 9, 651 10.3390/ma9080651. 28773773 PMC5509262

[ref20] Ta T. D.; Ta H. D.; Tieu K. A.; Tran B. H. Impact of Chosen Force Fields and Applied Load on Thin Film Lubrication. Friction 2021, 9, 1259 10.1007/s40544-020-0464-2.

[ref21] Lacks D. J. Energy Landscapes and the Non-Newtonian Viscosity of Liquids and Glasses. Phys. Rev. Lett. 2001, 87, 225502 10.1103/PhysRevLett.87.225502. 11736406

[ref22] Lin L.; Kedzierski M. A. Density and Viscosity of a Polyol Ester Lubricant: Measurement and Molecular Dynamics Simulation. International Journal of Refrigeration 2020, 118, 188–201. 10.1016/j.ijrefrig.2020.07.004. PMC7918279 33654333

[ref23] Maginn E. J.; Messerly R. A.; Carlson D. J.; Roe D. R.; Elliott J. R. Best Practices for Computing Transport Properties 1. Self-Diffusivity and Viscosity from Equilibrium Molecular Dynamics [Article v1.0]. LiveCoMS 2019, 1 (1), 6324 10.33011/livecoms.1.1.6324.

[ref24] Ewen J. P.; Heyes D. M.; Dini D. Advances in Nonequilibrium Molecular Dynamics Simulations of Lubricants and Additives. Friction 2018, 6, 349–386. 10.1007/s40544-018-0207-9.

[ref25] Todd B. D.; Daivis P. J. Homogeneous Non-Equilibrium Molecular Dynamics Simulations of Viscous Flow: Techniques and Applications. Mol. Simul. 2007, 33, 189–229. 10.1080/08927020601026629.

[ref26] Jabbarzadeh A.; Tanner R. I. Molecular Dynamics Simulation and Its Application to Nano-Rheology. Rheology Reviews 2006, 52, 165-216.

[ref27] Zhang J.; Tan A.; Spikes H. Effect of Base Oil Structure on Elastohydrodynamic Friction. Tribol Lett. 2017, 65, 13 10.1007/s11249-016-0791-7.

[ref28] Yamawaki H. Viscosity Measurements of High-Pressure Liquids via a Quartz Crystal Fundamental Resonance. J. Appl. Phys. 2020, 127, 094701 10.1063/1.5143161.

[ref29] Yamawaki H. Pressure Dependence of Bis(2-Ethylhexyl) Sebacate and VG32 Hydraulic Oil Viscosities Using a Quartz Crystal Resonator. Int. J. Thermophys 2018, 39, 98 10.1007/s10765-018-2419-7.

[ref30] Wu Y.; Li W.; Wang X. The Influence of Oxidation on the Tribological Performance of Diester Lubricant. Lubr. Sci. 2014, 26, 55–65. 10.1002/ls.1229.

[ref31] Qian X.; Xiang Y.; Shang H.; Cheng B.; Zhan S.; Li J. Thermal-Oxidation Mechanism of Dioctyl Adipate Base Oil. Friction 2016, 4, 29–38. 10.1007/s40544-015-0099-x.

[ref32] Puscas C.; Bandur G.; Modra D.; Nutiu R. Mixtures of Vegetable Oils and Di-2-Ethylhexyl-Sebacate as Lubricants. J. Synth. Lubr. 2006, 23, 185–196. 10.1002/jsl.21.

[ref33] Comuñas M. J. P.; Bazile J.-P.; Lugo L.; Baylaucq A.; Ferńandez J.; Boned C. Influence of the Molecular Structure on the Volumetric Properties and Viscosities of Dialkyl Adipates (Dimethyl, Diethyl, and Diisobutyl Adipates). J. Chem. Eng. Data 2010, 55, 3697–3703. 10.1021/je100237h.

[ref34] Ewen J. P.; Gattinoni C.; Zhang J.; Heyes D. M.; Spikes H. A.; Dini D. On the Effect of Confined Fluid Molecular Structure on Nonequilibrium Phase Behaviour and Friction. Phys. Chem. Chem. Phys. 2017, 19, 17883–17894. 10.1039/C7CP01895A. 28660933

[ref35] Mundy C. J.; Balasubramanian S.; Bagchi K.; Siepmann J. I.; Klein M. L. Equilibrium and Non-Equilibrium Simulation Studies of Fluid Alkanes in Bulk and at Interfaces. Faraday Discuss. 1996, 104, 17–36. 10.1039/fd9960400017.

[ref36] Kondratyuk N. D.; Pisarev V. V.; Ewen J. P. Probing the High-Pressure Viscosity of Hydrocarbon Mixtures Using Molecular Dynamics Simulations. J. Chem. Phys. 2020, 153, 154502 10.1063/5.0028393. 33092386

[ref37] Verlet L. Computer ”Experiments” on Classical Fluids. I. Thermodynamical Properties of Lennard-Jones Molecules. Phys. Rev. 1967, 159, 98–103. 10.1103/PhysRev.159.98.

[ref38] Kubo R. Statistical-Mechanical Theory of Irreversible Processes. I. General Theory and Simple Applications to Magnetic and Conduction Problems. J. Phys. Soc. Jpn. 1957, 12, 570–586. 10.1143/JPSJ.12.570.

[ref39] Todd B. Computer Simulation of Simple and Complex Atomistic Fluids by Nonequilibrium Molecular Dynamics Techniques. Comput. Phys. Commun. 2001, 142, 14–21. 10.1016/S0010-4655(01)00304-6.

[ref40] Todd B. D.; Daivis P. J. Nonequilibrium Molecular Dynamics: Theory, Algorithms and Applications; Cambridge University Press: Cambridge, 2017.

[ref41] Nosé S. A Molecular Dynamics Method for Simulations in the Canonical Ensemble. Mol. Phys. 1984, 52, 255–268. 10.1080/00268978400101201.

[ref42] Hoover W. G. Canonical Dynamics: Equilibrium Phase-Space Distributions. Phys. Rev. A 1985, 31, 1695–1697. 10.1103/PhysRevA.31.1695. 9895674

[ref43] Martínez L.; Andrade R.; Birgin E. G.; Martínez J. M. PACKMOL: A package for building initial configurations for molecular dynamics simulations. J. Comput. Chem. 2009, 30, 2157–2164. 10.1002/jcc.21224. 19229944

[ref44] Jewett A. I.; Stelter D.; Lambert J.; Saladi S. M.; Roscioni O. M.; Ricci M.; Autin L.; Maritan M.; Bashusqeh S. M.; Keyes T.; et al. Moltemplate: A Tool for Coarse-Grained Modeling of Complex Biological Matter and Soft Condensed Matter Physics. J. Mol. Biol. 2021, 433, 166841 10.1016/j.jmb.2021.166841. 33539886 PMC8119336

[ref45] Thompson A. P.; Aktulga H. M.; Berger R.; Bolintineanu D. S.; Brown W. M.; Crozier P. S.; in ’t Veld P. J.; Kohlmeyer A.; Moore S. G.; Nguyen T. D.; et al. LAMMPS - a Flexible Simulation Tool for Particle-Based Materials Modeling at the Atomic, Meso, and Continuum Scales. Comput. Phys. Commun. 2022, 271, 108171 10.1016/j.cpc.2021.108171.

[ref46] Pluhackova K.; Morhenn H.; Lautner L.; Lohstroh W.; Nemkovski K. S.; Unruh T.; Böckmann R. A. Extension of the LOPLS-AA Force Field for Alcohols, Esters, and Monoolein Bilayers and Its Validation by Neutron Scattering Experiments. J. Phys. Chem. B 2015, 119, 15287–15299. 10.1021/acs.jpcb.5b08569. 26537654

[ref47] Hockney R. W.; Eastwood J. W. Computer Simulation Using Particles; CRC Press: 1988.

[ref48] Bernardino K.; Ribeiro M. C. Pressure and shear rate effects on viscosity and structure of imidazolium-based ionic liquids. Fluid Phase Equilib. 2022, 554, 113345 10.1016/j.fluid.2021.113345.

