# Supplementary material for: Computing Viscosities of Mixtures of Ester-Based Lubricants at Different Temperatures
Source: J Phys Chem B. 2023 Mar 8;127(11):2587–94. doi: 10.1021/acs.jpcb.2c08553 (PMC10041636; doi:10.1021/acs.jpcb.2c08553)
Supplement: Supplementary file 1 — jp2c08553_si_001.pdf [file jp2c08553_si_001.pdf]

Computing Viscosities of Mixtures of Ester-Based Lubricants at Different Temperatures

Davide Sarpa[1], Dimitrios Mathas[1], Vasilios Bakolas[2], Joanna Procelewska[2], Joerg Franke[2], Martin Busch[2], Philipp Roedel[2], Christof Bohnert[2], Marcus Wolf[2], and Chris-Kriton Skylaris[1]

[1]Department of Chemistry, University of Southampton, Highfield, University Road, Southampton SO17 1BJ, UK

[2]Schaeffler Technologies AG & Co. KG, Industriestraße 1-3 91074, Herzogenaurach, Germany

February 23, 2023

Table S1: Number of molecules per mixture

Mixture	DEHS	DEHA
100% DEHS	150	0
90% DEHS	135	17
80% DEHS	121	35
70% DEHS	106	52
60% DEHS	91	70
50% DEHS	76	87
40% DEHS	61	105
30% DEHS	46	123
20% DEHS	31	141
10% DEHS	15	158
0% DEHS	0	176

Table S2: Experimental densities in g/cm^3 at 293 K

Mixture	Measurement 1	Measurement 2	Measurement 3
100% DEHS	0.9141	0.9141	0.9142
90% DEHS	0.9152	0.9140	0.9142
80% DEHS	0.9163	0.9153	0.9154
70% DEHS	0.9170	0.9167	0.9165
60% DEHS	0.9153	0.9187	0.9183
50% DEHS	0.9151	0.9186	0.9186
40% DEHS	0.9194	0.9211	0.9210
30% DEHS	0.9205	0.9226	0.9227
20% DEHS	0.9213	0.9232	0.9232
10% DEHS	0.9207	0.9242	0.9241
0% DEHS	0.9253	0.9255	0.9254

Table S3: Experimental densities in g/cm^3 at 343 K

Mixture	Measurement 1	Measurement 2	Measurement 3
100% DEHS	0.8782	0.8781	0.8778
90% DEHS	0.8792	0.8780	0.8782
80% DEHS	0.8800	0.8791	0.8791
70% DEHS	0.8802	0.8816	0.8811
60% DEHS	0.8754	0.8814	0.8812
50% DEHS	0.8715	0.8826	0.8825
40% DEHS	0.8810	0.8838	0.8838
30% DEHS	0.8809	0.8847	0.8847
20% DEHS	0.8824	0.8857	0.8858
10% DEHS	0.8782	0.8868	0.8868
0% DEHS	0.8878	0.8877	0.8877

Table S4: Experimental densities in g/cm^3 at 393 K

Mixture	Measurement 1	Measurement 2	Measurement 3
100% DEHS	0.8425	0.84216	0.84205
90% DEHS	0.8433	0.84215	0.84148
80% DEHS	0.8441	0.84295	0.84278
70% DEHS	0.8444	0.84447	0.84467
60% DEHS	0.8385	0.84618	0.84594
50% DEHS	0.8322	0.84723	0.84697
40% DEHS	0.8444	0.84685	0.84685
30% DEHS	0.8426	0.84743	0.84743
20% DEHS	0.8473	0.84844	0.84846
10% DEHS	0.8392	0.84922	0.84925
0% DEHS	0.8505	0.84743	0.85014

Table S5: Experimental viscosities in mPa · s at 293 K

Mixture	Measurement 1	Measurement 2	Measurement 3
100% DEHS	21.4	21.587	21.582
90% DEHS	20.5	21.442	21.493
80% DEHS	19.6	20.500	20.550
70% DEHS	18.8	19.545	19.707
60% DEHS	17.9	18.103	18.229
50% DEHS	17.1	17.948	18.045
40% DEHS	16.4	16.314	16.363
30% DEHS	15.6	15.358	15.415
20% DEHS	15.0	14.941	14.979
10% DEHS	14.4	14.395	14.431
0% DEHS	13.7	13.714	13.739

Table S6: Experimental viscosities in mPa · s at 343 K

Sample	Measurment 1	Measurement 2	Measurement 3
100% DEHS	4.80	4.8956	4.8855
90% DEHS	4.69	4.8931	4.8844
80% DEHS	4.53	4.7253	4.7167
70% DEHS	4.37	4.3075	4.3222
60% DEHS	4.23	4.3410	4.3642
50% DEHS	4.07	4.1514	4.1640
40% DEHS	3.94	3.9736	3.9676
30% DEHS	3.78	3.8380	3.8314
20% DEHS	3.67	3.6894	3.6821
10% DEHS	3.55	3.5453	3.5389
0% DEHS	3.42	3.4364	3.4320

Table S7: Experimental viscosities in mPa · s at 393 K

Mixture	Measurement 1	Measurement 2	Measurement 3
100% DEHS	2.05	2.1025	2.0930
90% DEHS	2.02	2.0993	2.0930
80% DEHS	1.96	2.0342	2.0311
70% DEHS	1.90	1.9146	1.9202
60% DEHS	1.85	1.8084	1.8185
50% DEHS	1.79	1.7356	1.7449
40% DEHS	1.74	1.7560	1.7567
30% DEHS	1.68	1.7157	1.7130
20% DEHS	1.63	1.6513	1.6468
10% DEHS	1.59	1.5980	1.5954
0% DEHA	1.54	1.5478	1.5460

Table S8: NEMD Simulated viscosities in mPa · s at 393 K using a shear rate of $10^{8.0}$ s^-1

Mixture	Sim 1	Sim 2	Sim 3	Sim 4	Sim 5
100% DEHS	1.47567	1.93383	2.29641	2.61061	2.06284
90% DEHS	1.90497	1.38599	1.64744	1.2129	0.840531
80% DEHS	1.53953	1.9249	0.923076	1.74947	1.24904
70% DEHS	1.09814	1.4156	0.892657	1.49023	2.67313
60% DEHS	1.56766	0.999813	1.82259	1.54681	1.82386
50% DEHS	1.44349	2.09275	1.34165	1.05732	1.26873
40% DEHS	1.88127	1.30093	2.21765	1.19368	1.79214
30% DEHS	1.41055	0.456006	1.22551	1.43333	1.19204
20% DEHS	1.75655	1.29741	0.837279	1.73486	1.23938
10% DEHS	0.637257	1.4794	1.86702	0.886978	1.38416
0% DEHS	1.31866	1.26602	0.751429	1.32701	1.63518

Table S9: EMD Simulated viscosities in mPa · s at 393 K

Mixture	Sim 1	Sim 2	Sim 3	Sim 4	Sim 5
100% DEHS	1.35681	1.37317	1.54107	1.44738	1.56535
90% DEHS	1.56489	1.44647	1.45993	1.57633	1.4598
80% DEHS	1.33518	1.45534	1.5224	1.45473	1.38451
70% DEHS	1.36545	1.33665	1.29309	1.47255	1.3753
60% DEHS	1.20986	1.31837	1.25017	1.44903	1.39569
50% DEHS	1.15665	1.42013	1.27373	1.24578	1.17834
40% DEHS	0.979914	1.51036	1.37516	1.31516	1.14249
30% DEHS	1.13143	1.25615	1.27258	1.4375	1.13405
20% DEHS	1.15201	1.23664	1.38756	1.13892	1.22566
10% DEHS	1.3194	1.21002	1.13747	1.10126	1.29524
0% DEHS	1.33386	1.23307	1.17444	1.09871	1.1533

Table S10: NEMD Simulated viscosities in mPa · s at 343 K using a shear rate of $10^{8.0}$ s^-1

Mixture	Sim 1	Sim 2	Sim 3	Sim 4	Sim 5
100% DEHS	5.20373	3.17659	2.55105	3.86292	4.08831
90% DEHS	3.74814	4.34317	3.7022	3.82062	4.06491
80% DEHS	3.04861	3.98753	4.11072	2.63283	4.06506
70% DEHS	3.79686	1.86459	1.61644	3.33484	3.07929
60% DEHS	2.85277	2.68636	3.62504	2.1453	2.2235
50% DEHS	1.6346	3.46118	1.94045	3.68776	3.29252
40% DEHS	3.94019	2.93982	4.12112	2.80574	4.77117
30% DEHS	3.68474	3.35464	3.88528	3.94409	3.55292
20% DEHS	3.08243	3.17085	3.33484	2.43976	1.93192
10% DEHS	2.44014	3.3542	2.97634	3.71123	3.0543
0% DEHS	2.94679	3.01567	2.24485	4.08723	3.90481

Table S11: NEMD Simulated viscosities in $mPa \cdot s$ at 293 K using a shear rate of $10^{8.0}$ s^-1

Mixture	Sim 1	Sim 2	Sim 3	Sim 4	Sim 5
100% DEHS	19.3601	19.0723	20.7817	17.7328	19.7875
90% DEHS	17.309	17.1312	18.9698	16.2492	16.8455
80% DEHS	12.7746	20.3967	16.7248	17.43	16.9771
70% DEHS	18.09	12.4066	15.7088	16.7124	13.9906
60% DEHS	15.4708	13.2387	15.0742	15.5904	16.5427
50% DEHS	16.895	14.0516	13.4636	14.8098	13.4153
40% DEHS	11.6325	15.3372	15.0181	14.2087	12.3551
30% DEHS	11.8026	12.9843	15.037	14.1295	12.7197
20% DEHS	12.8643	12.8756	13.2167	13.9933	11.5103
10% DEHS	9.06055	13.7538	12.6618	12.3689	12.6936
0% DEHS	11.3143	11.7982	12.071	12.7719	11.5679
